# Impact of Dara-VTD induction therapy on stem cell mobilization outcomes in newly diagnosed multiple myeloma patients undergoing autologous stem cell transplantation: a multicenter study

**DOI:** 10.1007/s00277-025-06581-x

**Published:** 2025-09-05

**Authors:** Roberta Della Pepa, Salvatore Palmieri, Stefano Rocco, Novella Pugliese, Aldo Leone, Simona Avilia, Marialucia Barone, Rosa Rosamilio, Fabio Trastulli, Danilo De Novellis, Raffaele Fontana, Bianca Serio, Denise Morini, Lorenzo Esposito, Laura De Fazio, Roberta Spisso, Carmine Selleri, Catello Califano, Alessandra Picardi, Mario Annunziata, Fabrizio Pane

**Affiliations:** 1 Hematology Unit, Department of Clinical Medicine and Surgery, University of Naples “Federico II”, Naples, Italy; 2 Division of Hematology, AO “A. Cardarelli’’ Hospital, Naples, Italy; 3 Onco-Hematology Unit, “A. Tortora” Hospital, Pagani, Italy; 4 Hematology and Transplant Center, University Hospital “San Giovanni Di Dio E Ruggi d’Aragona”, Salerno, Italy; 5 Division of Hematology and SCT Unit, AO “A. Cardarelli” Hospital, Naples, Italy

**Keywords:** Multiple myeloma, Dara-VTD, Stem cell mobilization, ASCT

## Abstract

Daratumumab combined with bortezomib, thalidomide, and dexamethasone (Dara-VTD) is a highly effective induction therapy for newly diagnosed multiple myeloma (NDMM) patients eligible for autologous stem cell transplantation (ASCT). However, its impact on stem cell mobilization requires a critical evaluation. This study examines the effects of Dara-VTD on stem cell mobilization and collection outcomes. A multicenter retrospective study included 81 consecutive NDMM patients treated with Dara-VTD (from November 2021 to June 2023). Data on stem cell mobilization and collection were compared with 93 historical VTD patients. Mobilization regimens included cyclophosphamide (CTX), vinorelbine + CTX, and chemotherapy-free approaches, with plerixafor used as rescue therapy. Mobilization success was evaluated by CD34 + cell yield, additional agent use, and leukapheresis sessions required. The median CD34 + yield in the Dara-VTD group was 5.1 million cells/kg, with 96.3% of patients achieving > 2 × 10^6 cells/kg of body weight. Plerixafor use was significantly higher in the Dara-VTD group (56.2%) compared to VTD (4.3%), and CTX-based regimens showed superior mobilization efficacy (*p* = 0.01). Engraftment was faster in the Dara-VTD group, with median neutrophil and platelet recovery at 11 and 13 days, compared to 12 and 17 days in the VTD group (*p* < 0.05). Dara-VTD maintains the feasibility of ASCT, with comparable stem cell mobilization and collection outcomes to VTD. Mobilization success is influenced by individualized strategies, with CTX and plerixafor playing key roles in optimizing stem cell yield. Despite the challenges posed by daratumumab, stem cell mobilization remains effective, and Dara-VTD does not compromise the transplant process.

## Background

Dara-VTD has demonstrated significant efficacy as an induction therapy for patients with NDMM who are eligible for ASCT. In the registrative trial Cassiopeia this regimen proved enhanced remission rates and improved progression-free survival when compared to the standard arm VTD [1]. However, its impact on hematopoietic stem cell collection is a critical consideration [2–5]. The addition of daratumumab to the traditional VTD protocol has been associated with deeper remissions [1] but may also complicate stem cell mobilization. Specifically, data from GRIFFIN trial [6] suggest that while Dara-VTD is effective, it can reduce the yield of CD34 + stem cells, which are essential for successful ASCT. To minimize potential mobilization challenges, the use of granulocyte colony-stimulating factor (G-CSF) alone or in combination with chemomobilization agents like CTX is common [4]. Plerixafor, a CXCR4 inhibitor, is also often employed as a rescue therapy for poor mobilizers or preemptively in high-risk cases [7]. Successful peripheral blood stem cell (PBSC) harvest remains a key factor for ASCT, with a minimum number of 2.0 × 10^6 CD34 +/kg required for a single procedure. The International Myeloma Working Group has suggested a minimum target of 4 × 10^6 CD34 +/kg and, if feasible, an average of 8 − 10 × 10^6 CD34 +/kg should be collected, allowing most myeloma patients to undergo two autografts during the course of their disease [8]. Thus, while Dara-VTD improves clinical outcomes, careful management of stem cell mobilization and collection is essential to ensure the success of subsequent ASCT.

This retrospective study aimed to analyze the outcomes of hematopoietic stem cell mobilization and collection in 81 consecutive patients with NDMM who underwent Dara-VTD induction therapy before hematopoietic stem cell mobilization, high-dose chemotherapy, and ASCT.

## Aims and methods

This multi-center retrospective analysis included patients with NDMM who had received induction therapy with Dara-VTD and were candidates for upfront high-dose chemotherapy and ASCT. Our objective was to investigate the efficacy and impact of the Dara-VTD induction therapy regimen on subsequent stem cell mobilization and collection, essential steps in the treatment of patients with NDMM. Clinical data for the Dara-VTD cohort were collected from November 2021 to June 2023 across four hospitals in the Campania Region of Italy. We compared various mobilization regimens within the Dara-VTD cohort, aiming to identify significant differences in stem cell yield, the necessity for additional mobilization agents like plerixafor, and other key metrics such as the number of leukapheresis sessions required. We also compared stem cell yield and engraftment data with a historical cohort treated with VTD. Mobilization regimens were applied to all patients, comprising: CTX (3 g/mq day 1) plus granulocyte G-CSF at a dose of 10 µg/kg/day from day 4 until sufficient peripheral blood (PB) CD34 + cell counts or day + 7 [4]; vinorelbine (25 mg/mq day 1) and CTX (1,5 g/mq day 2), plus G-CSF at a dose of 10 µg/kg/day from day 3 until sufficient PB CD34 + cell counts or day + 7 [9]; chemo-free regimen with only G-CSF at a dose of 10 µg/kg/day from day 1 until sufficient PB CD34 + cell counts or day + 7 [10].

Leukoapheresis was initiated if the PB CD34 + cell count exceeded 10/μL. According to institutional policies, plerixafor was always given if the collection goal (2.0 × 10^6 CD34 + cells/kg) was not met, but at the provider’s discretion. Peripheral venous access was used for apheresis in the majority of cases. Central venous access (temporary femoral catheter) was reserved for patients with inadequate peripheral veins.

Patients were classified as good mobilizers, poor mobilizers, or non-mobilizers based on their ability to collect CD34 + cells. A good mobilizer successfully collects the required threshold of CD34 + cells, typically meeting or exceeding 2 × 10^6 cells/kg of body weight during apheresis. A poor mobilizer, on the other hand, fails to reach this threshold, collecting fewer than the required cells despite undergoing mobilization therapies, often necessitating multiple apheresis sessions or additional interventions. A non-mobilizer is characterized by an inability to collect any viable CD34 + cells during apheresis, with cell counts falling below detectable levels.

Data were collected in spreadsheets and analyzed using R statistical software (v. 4.0.5; RStudio). Differences between groups were assessed by chi-square, Fisher's, Wilcoxon signed-rank, or unpaired two-tailed t-tests. A p value of < 0.05was considered statistically significant.

## Results

### Patient characteristics

The baseline demographic and clinical characteristics of the Dara-VTD group (*n* = 81) are summarized in Table 1. In the Dara-VTD cohort (n = 81), 47% of the patients were female, with a median age of 59 years. Most patients had the IgG subtype (62.5%), while 10% presented with light chain-only disease and 1.2% with nonsecretory myeloma. According to the International Staging System (ISS), 48.1% were classified as stage I, 30.9% as stage II, and 21% as stage III. The Revised ISS (R-ISS) classification indicated 28.4% of patients in stage I, 58% in stage II, and 12.3% in stage III.

**Table 1:** Baseline demographic and clinical characteristics of Dara-VTD and VTD groups

Variable	DARA-VTD (*n* = 81)	VTD (*n* = 93)	*P* value
Sex			
Female	38 (47)	46 (40.5)	0.85
Male	43 (53)	47 (49.5)	
Age, years			
Median, (range)	59	57.5	0.17
Subtype			
IgG	50 (62.5)	59 (63.5)	0.13
IgA	21 (26.3)	14 (15)	
Light chain only	8 (10)	16 (17.2)	
Nonsecretory	1 (1.2)	4 (4.3)	
ISS stage			
I	39 (48.1)	44 (64.7)	0.086
II	25 (30.9)	17 (25)	
III	17 (21)	7 (10.3)	
Unknown	0	25 (26.9)	
R-ISS stage			
I	23 (28.4)	6 (6.5)	0.87
II	47 (58)	10 (10.8)	
III	10 (12.3)	3 (3.2)	
Unknown	0	74 (78.6)	
Mobilization therapy			
Cyclophosphamide	23 (28.4)	13 (14)	< 0.0001
Vinorelbine + Cyclophosphamide	27 (33.3)	78 (83.8)	
CHT free	31 (38.3)	2 (2.2)	
Use of plerixafor			
Yes	45 (56.2)	4 (4.3)	< 0.0001
CD34 + cells collected (× 10^6^ cells/kg)			
Median (range)	5.1 (0–16.8)	5.4 (0–17.85)	0.49
Apheresis days			
Median (range)	2 (1–3)	1 (1–2)	0.0022
Mobilization status			
Good	78 (96.3)	92 (98.9)	0.43
Poor	1 (1.2)	0	
No	2 (2.4)	1 (0.1)	
Disease status prior to ASCT			
CR	21 (25.9)	26 (27.9)	0.066
VGPR	55 (67.9)	48 (51.6)	
PR	4 (4.9)	13 (13.9)	
SD	0	2 (2.1)	
PD	1 (1.2)	4 (4.3)	

*DARA-VTD* Daratumumab, bortezomib, thalidomide, dexamethasone, *VTD* Bortezomib, thalidomide, dexamethasone, *ISS* International Staging System; R-ISS, Revised-ISS, *CHT* Chemotherapy, *ASCT* Autologous stem cell transplant, *CR* Complete response, *VGPR* Very good partial response, *PR* Partial response, *SD* Stable response, *PD* Progressive disease

In comparison with the VTD group (*n* = 93), several differences emerged (Table 1): the VTD group had a higher percentage of light chain-only cases (17.2% versus 10% in Dara-VTD) and a slightly greater incidence of nonsecretory myeloma (4.3% versus 1.2% in Dara-VTD), *p* = 0.13. Regarding ISS staging, stage I was more common in the VTD group (64.7% compared to 48.1% in Dara-VTD, *p* = 0.086). Lastly, in the R-ISS, stage I was less represented in the VTD group (6.5%) compared to the Dara-VTD group (28.4%), *p* = 0.87.

### Disease status prior to ASCT

In the Dara-VTD group, 25.9% of patients achieved a complete response (CR) before ASCT, while the majority (67.9%) attained a very good partial response (VGPR). Only 4.9% of patients reached a partial response (PR), reflecting a generally strong treatment response. In comparison, the VTD group showed similar rates of CR (27.9%) but had a lower percentage of patients achieving VGPR (51.6%) and a higher percentage with PR (13.9%). These differences were not statistically significant (*p* = 0.43).

### Mobilization therapy

In the Dara-VTD group, mobilization therapy varied, with 28.4% of patients receiving CTX as a single agent. Additionally, 33.3% of patients were mobilized with a combined regimen of Vinorelbine and CTX. Notably, a considerable portion of patients in the Dara-VTD group (38.3%) were classified as chemotherapy-free (CHT-free). In contrast, the VTD group had a significantly lower use of CTX alone, with only 14% of patients receiving this regimen (*p* < 0.0001). The majority of VTD patients (83.8%) were mobilized using the combined Vinorelbine and CTX regimen, showing a strong preference for dual-agent mobilization in this group. Only 2.2% of patients in the VTD group were managed with a chemotherapy-free approach.

### Plerixafor use

All patients received G-CSF for mobilization, while the use of plerixafor was notably higher in the Dara-VTD cohort, with 56.2% receiving it [median of 1 dose (range: 1–3)] compared to 4.3% in the VTD group (*p* < 0.0001).

### PBSC mobilization metrics

The average interval between the last day of induction therapy and the first day of stem cell collection was 14 days (range: 6–36 days) in the Dara-VTD group and 15 days (range: 7–40 days) in the VTD group. In the Dara-VTD group, the median CD34 + cell yield was 5.1 million cells/kg (range: 0–16.8), with a median apheresis duration of 2 days (range: 1–3). Good mobilization status was achieved by 96.3% of patients, with 96.3% collecting more than 2 × 10^6 cells/kg, 1.2% showing poor mobilization (< 2 × 10^6 cells/kg), and 2.4% failing to mobilize. Among patients in the Dara-VTD group, bone marrow plasma cell infiltration at diagnosis was available in 74/81 patients (median 52%, range: 10–90%). A trend toward lower CD34 + yields was observed in patients with higher baseline infiltration (> 60%) compared to those with < 60%, although this did not reach statistical significance (*p* = 0.08).

**Fig. 1:**
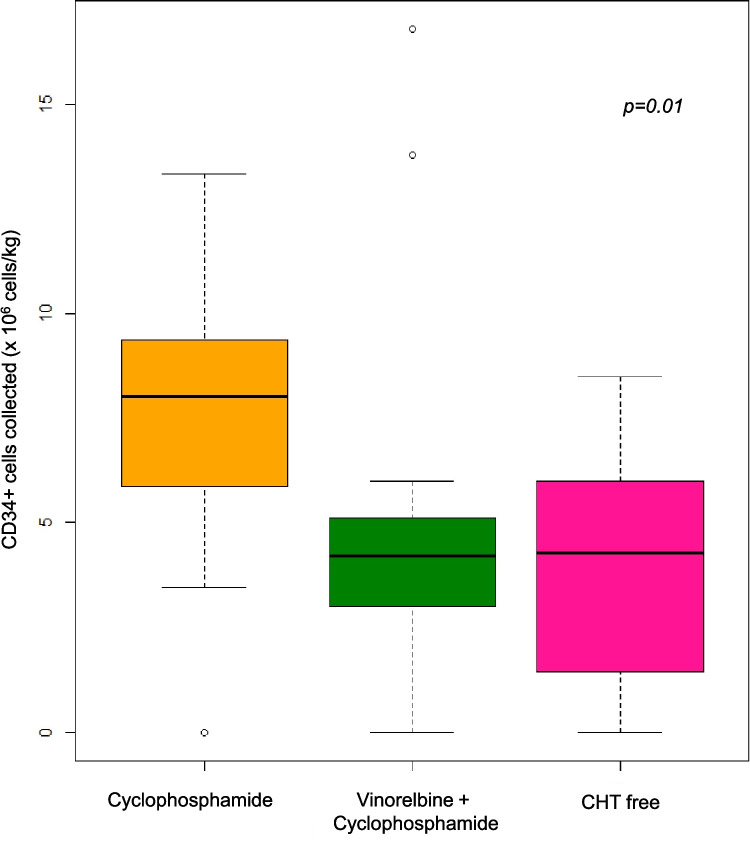
Comparison of CD34 + cell yields in the Dara-VTD group among patients treated with CTX and G-CSF versus those receiving Vinorelbine + CTX or chemotherapy-free regimens

The yields varied across mobilization regimens: patients receiving CTX had a median CD34 + yield of 8.0 million cells/kg (range: 0–13.8), those with Vinorelbine + CTX had a median of 4.3 million cells/kg (range: 2.0–16.8), and the chemotherapy-free group collected 5.2 million cells/kg (range: 0–8.5). Additionally, stem cell collection efficacy was significantly associated with ISS stage at diagnosis (*p* = 0.048), and patients treated with CTX and G-CSF had notably higher CD34 + yields than those receiving Vinorelbine + CTX or chemotherapy-free regimens (*p* = 0.01, Fig. 1). The introduction of plerixafor enhanced collection quality significantly (*p * = 0.007), especially in patients mobilized with CTX or chemotherapy-free regimens. The median time from induction to apheresis was 2.03 months (range: 0.1–19.6).

**Fig. 2:**
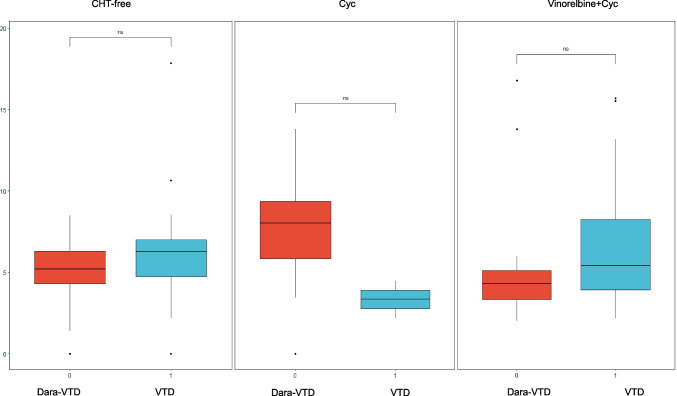
Mobilization status comparison between the Dara-VTD and VTD groups

In the VTD group, the median CD34 + yield was slightly higher at 5.4 million cells/kg (range: 0–17.85), with a shorter median apheresis duration (1 day, range 1–2) comparing with the Dara-VTD group (*p* < 0.05). Good mobilization was achieved by 98.9% of patients, with none classified as poor mobilizers and only 1.1% failing to mobilize. Statistical analysis revealed no significant difference between Dara-VTD and VTD groups regarding mobilization status, with a p-value of 0.43. (Fig. 2). For the different mobilization regimens, median yields were 3.34 million cells/kg (2.2–4.48) for CTX, 5.4 million cells/kg (2.2–15.7) for vinorelbine + CTX, and 6.3 million cells/kg (0–17.85) for chemotherapy-free regimens. The interval between the end of induction and apheresis was a median of 2 months (range: 0.32–62.7), with no significant differences in mobilization status or efficiency compared to the Dara-VTD group (*p* = 0.43). The use of plerixafor, as demonstrated in the Dara-VTD group, significantly enhanced stem cell collection quality across all mobilization regimens (*p* = 0.007), with a higher proportion of patients receiving plerixafor in the CTX and chemotherapy-free groups. The median time from the end of induction therapy to apheresis was 2 months (range: 0.32–62.7) in this group.

### Engraftment

In the Dara-VTD group, the median time to neutrophil engraftment was 11 days (range: 9–16), while platelet engraftment occurred at a median of 13 days (range: 11–18). In the VTD group, the median time to neutrophil engraftment was slightly longer at 12 days (range: 7–24), and platelet engraftment occurred at a median of 17 days (range: 20–26). Both of these times were significantly slower compared to the Dara-VTD group, with p-values of 0.044 and 0.0048, respectively.

## Discussion

Despite the advent of novel therapies, ASCT remains a fundamental treatment modality in the first-line management of Multiple myeloma (MM) for eligible patients, particularly in achieving deep remissions [1]. However, the process of mobilizing CD34 + and CD38 + hematopoietic stem cells (HSCs) from the bone marrow to peripheral blood—a crucial step for successful ASCT—can be challenging [2, 3, 5].

Tandem transplants (double ASCT) have historically been a common approach for high-risk MM patients, especially in those patients with high risk cytogenetic alterations, aggressive disease or suboptimal responses to induction therapy [11]. However, with the advent of potent induction quadruplet regimens like Dara-VTD and lenalidomide maintenance, the necessity of double ASCT is being increasingly questioned [12–14]. These therapies often achieve deeper remissions, including higher rates of minimal residual disease (MRD) negativity [15]. As a result, the clinical benefit of collecting CD34 + stem cells for two or more transplants is less clear in this setting. In this view, it may no longer be mandatory to target CD34 + cell collection for two transplants, as single ASCT combined with these potent therapies can often deliver durable responses. This shift could help reduce the burden on patients and streamline the mobilization process without compromising treatment efficacy.

Factors such as advanced disease stage, extensive prior therapy, and the inclusion of certain agents in induction regimens, may negatively impact stem cell mobilization. daratumumab has been associated with complications in stem cell collection due to its potential effects on bone marrow plasmacells, which might impair the release of CD34 + cells into the bloodstream [16]. Several biological mechanisms have been proposed to explain the reduced mobilization of CD34 + cells following daratumumab-based induction therapy. First, daratumumab is an anti-CD38 monoclonal antibody that targets CD38, a glycoprotein expressed on the surface of plasma cells and other immune cells, including hematopoietic progenitor cells and bone marrow stromal cells. CD38 plays a role in cellular adhesion, migration, and signaling within the bone marrow microenvironment. By binding to CD38, daratumumab may disrupt these interactions, impairing the ability of hematopoietic stem cells (HSCs) to detach from the bone marrow niche and migrate into the bloodstream. This disruption could hinder the effective mobilization of CD34 + cells, which are critical for successful stem cell collection [17]. Furthermore, the bone marrow niche plays a key role in maintaining HSCs in a quiescent state. Daratumumab's impact on the bone marrow microenvironment, particularly its depletion of regulatory cells like plasma cells and other CD38-expressing cells, may alter the balance of cytokines and chemokines required for efficient stem cell release. Specifically, it has been suggested that daratumumab might reduce the levels of certain cytokines that promote the egress of CD34 + cells, such as stromal cell-derived factor 1 (SDF-1), which is critical for HSC mobilization [18]. Additionally, daratumumab induces antibody-dependent cellular cytotoxicity and complement-dependent cytotoxicity of CD38-expressing cells, leading to the depletion of various immune cells. This includes not only plasma cells but also natural killer cells and regulatory T cells. These cells play a role in maintaining homeostasis within the bone marrow microenvironment. Their depletion may further impair the mobilization process by reducing support for HSC proliferation and trafficking from the bone marrow to peripheral blood [19]. Finally, although CD34 + stem cells are not highly expressed with CD38 in the same manner as mature plasma cells, there is evidence that CD38 may still be present in certain subsets of progenitor cells. The direct targeting of these subsets by daratumumab could interfere with their functional capacity to mobilize into the bloodstream, further contributing to the reduction in mobilized CD34 + cell counts [19].

The results of our analysis highlight significant insights into the efficacy of PBSC mobilization in MM patients undergoing ASCT after the quadruplet Dara-VTD induction. The median collection of CD34 + cells at 5 × 10^6 cells/kg, with a majority of patients (83.2%) achieving the target mobilization threshold of over 2 × 10^6 cells/kg, underscores the overall effectiveness of the mobilization strategies employed. However, the observed range in collected cells (0.57–16.8 × 10^6 cells/kg) indicates variability in mobilization success, which may be influenced by individual patient factors, such as disease stage and previous therapies. The significant correlation between stem cell collection efficacy and the ISS stage at diagnosis (*p* = 0.048) aligns with previous literature that emphasizes the role of disease stage in mobilization outcomes. Advanced disease stages can lead to suboptimal mobilization, making careful patient selection and monitoring critical in planning for ASCT [20]. This suggests that advanced disease stage, reflecting a more aggressive disease burden and compromised bone marrow reserve, plays a significant role in hindering effective stem cell mobilization. Furthermore, we observed that patients who received CTX as part of their mobilization regimen had superior stem cell yields compared to those on alternative protocols (*p* = 0.01). This finding, consistent with the results of the CASSIOPEIA trial, suggests that CTX, especially in high-risk patients, is a highly effective mobilization agent [1, 4].

Furthermore, the substantial impact of plerixafor on the quality of stem cell collection (*p* = 0.007) highlights its importance as an adjunctive therapy, particularly in patients at risk of poor mobilization. The increased use of plerixafor among those receiving CTX or chemotherapy-free regimens suggests its potential to mitigate mobilization challenges in these populations [21].

While CTX-based regimens demonstrated superior yields, the observation that chemotherapy-free mobilization achieved adequate collections in a substantial proportion of patients (38.3%) raises the question whether chemomobilization is always necessary in the context of potent quadruplet induction therapies. This warrants further prospective evaluation. The relatively high proportion of patients receiving CHT-free mobilization in the Dara-VTD group (38.3%) reflects an institutional preference aimed at minimizing treatment-related toxicity following intensive induction. Despite typically resulting in lower CD34 + yields, chemotherapy-free mobilization was considered appropriate for many patients, particularly those in deep remission or with relevant comorbidities.

Overall, our findings suggest a tailored approach to mobilization strategies, considering individual patient characteristics and the specific therapies employed, to optimize outcomes in MM patients undergoing ASCT.

Our data show that the outcomes of stem cell mobilization, engraftment, and the incidence of infectious events are comparable between the traditional VTD and Dara-VTD regimens, indicating that daratumumab does not compromise the transplant process. However, it is important to note a limitation in this comparison: while various mobilization regimens were employed in the Dara-VTD group as previously specified, a significant majority of patients in the historical VTD control group were mobilized using a vinorelbine-CTX regimen. This variation in mobilization strategies could influence the outcomes and interpretations of the data. Nonetheless, the findings suggest that the addition of daratumumab to the induction regimen not only supports effective stem cell mobilization but also does not compromise the safety or efficacy of the ASCT process. The significantly faster engraftment observed in the Dara-VTD group may be attributed to improved disease control prior to ASCT and possibly higher CD34 + cell doses infused in a subset of patients. Moreover, daratumumab-based induction may favor a more efficient hematopoietic niche repopulation, though this remains speculative and warrants further biological investigation. Importantly, Dara-VTD demonstrated non-inferiority in terms of key transplant-related outcomes, reinforcing its safety and efficacy as an induction regimen when compared to the conventional VTD protocol, as also supported by recent evidence [2, 5, 22]. The novelty of our work is limited: our findings are in line with previously published studies supporting the feasibility of stem cell collection following daratumumab-based induction, including data from Kwon et al.[23], Chhabra et al. [24], Mehl et al. [25], and Sauer et al. [26].

## Conclusions

Our retrospective multicenter study demonstrates that the introduction of daratumumab in the Dara-VTD regimen effectively maintains the feasibility of ASCT. Despite the known challenges associated with stem cell mobilization in the presence of daratumumab, our analysis reveals that the overall stem cell collection rates remain comparable to traditional VTD protocols, with a median collection of 5 × 10^6 CD34 + cells/kg and a high mobilization success rate across both cohorts.

The study highlights the critical role of tailored mobilization strategies, particularly the use of CTX in combination with G-CSF and adjunctive therapies like plerixafor, to optimize stem cell yields in the Dara-VTD cohort. Notably, our findings underscore the importance of patient-specific factors, such as disease stage and previous treatment history, in influencing mobilization outcomes. The significant correlation between mobilization efficacy and the ISS reinforces the necessity for careful patient assessment prior to ASCT.

While the results suggest that daratumumab does not compromise transplant safety or efficacy, further studies are warranted to refine mobilization protocols and to elucidate the underlying biological mechanisms influencing stem cell mobilization in this unique patient population. Overall, our findings contribute to the evolving landscape of MM treatment, emphasizing the need for continued research to enhance the success of ASCT and improve long-term outcomes for patients with NDMM.

## Data Availability

No datasets were generated or analysed during the current study.
